# “I Did My Own Research”: Overconfidence, (Dis)trust in Science, and Endorsement of Conspiracy Theories

**DOI:** 10.3389/fpsyg.2022.931865

**Published:** 2022-07-14

**Authors:** Andrea Vranic, Ivana Hromatko, Mirjana Tonković

**Affiliations:** Department of Psychology, Faculty of Humanities and Social Sciences, University of Zagreb, Zagreb, Croatia

**Keywords:** overconfidence, Dunning-Kruger effect, trust in science and scientists, conspiracy theories, conservatism

## Abstract

Epistemically suspect beliefs, such as endorsement of conspiracy theories or pseudoscientific claims, are widespread even among highly educated individuals. The phenomenon of conspiratorial thinking is not new, yet the COVID-19 pandemic, causing a global health crisis of an unprecedented scale, facilitated the emergence and rapid spread of some rather radical health-related pseudoscientific fallacies. Numerous correlates of the tendency to endorse conspiracy theories have already been addressed. However, many of them are not subject to an intervention. In this study, we have tested a model that includes predictors ranging from stable characteristics such as demographics (gender, age, education, and size of the place of residence), less stable general traits such as conservatism and overconfidence in one’s own reasoning abilities, to relatively changeable worldviews such as trust in science. A hierarchical regression analysis (*N* = 859 participants) showed that included predictors explained a total of 46% of the variance of believing in COVID-19 conspiracy theories, with only gender, overconfidence, and trust in science yielding significance. Trust in science was the strongest predictor, implying that campaigns aimed at enhancing public trust in both science as a process, and scientists as individuals conducting it, might contribute to the reduction in susceptibility to pseudoscientific claims. Furthermore, overconfidence in one’s own reasoning abilities was negatively correlated with an objective measure of reasoning (syllogisms test) and positively correlated with the endorsement of conspiracy theories, indicating that the so-called Dunning-Kruger effect plays a role in pseudoscientific conspiratorial thinking regarding COVID-19.

## Introduction

Conspiracies thrive in times of crisis. A reason behind this surge is the illusion of control, i.e., the soothing effect of reassuring oneself that troubles do not happen at random, but rather in a meaningful order. Endorsement of conspiracies occurs when official narratives are experienced as deficient and lip-deep, while events are viewed as deceitful, with non-conclusive explanations (Dagnall et al., 2017). Epistemically suspect beliefs are widespread and have far-reaching negative real-life outcomes. A conspiratorial worldview is linked to the refusal of science, the disregard for the biomedical model of disease, and legal means of political engagement (Imhoff and Lamberty, 2020).

Surveys worldwide show that a steady 20–30% of respondents believe that there is more to COVID-19 than meets the eye (e.g., Freeman et al., 2020; Pew Research Center, 2020). Regarding its origin, some “higher-order” types of conspiracies have been distinguished; it is either a man-made epidemic, created, and exaggerated for political reasons, or a hoax (with 49, 44, and 13% support, respectively; Frankovic, 2020). Their behavioral consequences are worrisome, with reduced guideline adherence and vaccination intentions being its most prominent individual and societal health hazards (for review, refer to Ripp and Röer, 2022). Conspiratorial beliefs have different displays; beliefs of the hoax are manifested as reduced guideline adherence, while those of a purposefully engineered virus are linked to increased self-serving preparatory behaviors (Imhoff and Lamberty, 2020).

Today’s society is characterized by a strong partisan division in terms of whom and what to believe in, and is faced with public mistrust in government, scientific endeavors, and medical/pharmaceutical corporations, as well as the media. Rebuilding this trust is a societal imperative. Research emerging within the inoculation theory (e.g., Jolley and Douglas, 2017; van der Linden et al., 2020) highlights that factual arguments can be effective in reducing early stage conspiracy belief before conspiratorial thinking has taken its root. Not surprisingly, studies are currently devoted to circumstances present prior to conspiracy taking its rise and to individual factors related to the tendency toward conspiratorial thinking.

### The Correlates of Conspiratorial Thinking

The variation in the degree to which individuals endorse conspiracy theories is attributable either to individual characteristics, such as demographics, personality, or one’s beliefs/attitudes, or to various social factors (group identity and trust in authorities). A recent review of 43 primary studies has highlighted individual variables related to the endorsement of COVID-19 conspiracies, yet overall results are sometimes conflicting (van Mulukom et al., 2022). While focusing on the correlates of conspiratorial thinking, we tackled the variables related to the potential of trust in science and its optimization in science communication, i.e., demographics, political orientation, and overconfidence.

#### Demographics

Studies often find that younger people are more inclined toward COVID-19 conspiracy beliefs, although opposite findings are evidenced in some cultures. While some find no gender differences in COVID-19-related conspiracy beliefs (Earnshaw et al., 2020; Freeman et al., 2020; Tonkoviæ et al., 2021), others find women more likely to fall prey to such beliefs. Aside from the possible cultural influences, in the case of COVID-19 conspiracy beliefs, gender effects seem to be overrun by personality tendencies such as learned helplessness and general conspiratorial thinking (van Mulukom et al., 2022). Education is also important, with earlier studies showing that lower education contributes to higher levels of conspiracy endorsement (e.g., Oliver and Wood, 2014a; Georgiou et al., 2020). However, some COVID-19 conspiracy theories were readily endorsed and dispersed even among highly educated individuals (Andrade, 2021; Ognyanova et al., 2021).

#### Conservatism

A reliable and consistent individual difference in generalized predispositions to endorse conspiracy theories seems to exist. Sutton and Douglas (2020) characterized it as a conspiracy mindset, even more so as a generalized political attitude. Conspiracy beliefs are found on both sides of the ideological spectrum, and general belief in conspiracies is strongest at both political extremes (van Prooijen et al., 2015). The threat caused by the COVID-19 pandemic has received different coverages by media platforms leaning toward conservative vs. liberal audiences (e.g., Druckman et al., 2021). Conservative media use predicts a higher rate of conspiracy beliefs, whereas reliance on mainstream media and print predicts their decrease (Romer and Jamieson, 2021). Conservatism is associated with lower perceptions of COVID-19 vulnerability and a stronger belief in both, the exaggerated impact of the virus put forth by the media and its spread viewed as a conspiracy. Furthermore, conservatives show less accurate knowledge of COVID-19 and do poor in disentangling real from fake news (e.g., Enders and Uscinski, 2021).

#### Overconfidence

While some individuals deliberately propagate misleading and partial information, some believe that any reasonable person, given enough study time and literature, is capable of insights regardless of the topic and expertise. Such superficial understanding of complex causal relations consequently leads to an overestimation of the quality and depth of one’s knowledge (Rozenblit and Keil, 2002)–a bias known as the Dunning-Kruger effect (DK effect; Kruger and Dunning, 1999). Often described as the “knowing less, presuming more,” DK effect explains poor decision-making and erroneous conclusions of unskilled individuals due to their metacognitive deficits and (in)competence in the field.

This inflated and unwarranted self-confidence might propagate conspiracy beliefs (Vitriol and Marsh, 2018). People generally believe to be more objective and less biased than their fellow citizens (e.g., Pronin et al., 2004). Also, they tend to overestimate their critical thinking capabilities (Lantian et al., 2020). Overconfidence in one’s attitudes and understanding tunes down the acceptance of knowledge and ideas across partisan boundaries and leads to polarization. In turn, it decreases the likelihood of encountering information, which might challenge or modify the existing beliefs and assumptions. Knowing less, but presuming more, might not be so worrisome with regard to COVID-19 had it not been staged in the era of social media where all the voices are equally loud; a unison scientific opinion is out-talked by an abundance of confident others.

#### Trust in Science

Understanding COVID-19 requires an understanding of complex biomedical mechanisms and dynamic statistical visualizations. Scientific communication on COVID-19 has changed alongside our understanding of the disease. The apparent inconsistencies have, in turn, spurred mistrust in scientists/science; COVID-19 conspiracies are often centered around the dismissal of scientific research (e.g., Tonkoviæ et al., 2021). Higher scientific enthusiasm is correlated with accurate knowledge and less misleading reasoning regarding COVID-19, whereas science skepticism is related to false beliefs and less support for the biomedical approach. Accordingly, mistrust of science is related to greater readiness to spread misinformation and lower guideline adherence (Brzezinski et al., 2020; Roozenbeek et al., 2020). The dismissal of scientific arguments is related to the self-reported knowledge of COVID-19 which is interpreted by denialism–a default mode of renunciation of expert opinions and a major predictor of the endorsement of conspiracy theories (Uscinski et al., 2020). van Mulukom et al. (2022) suggested that the belief in COVID-19 conspiracy theories may be boosted by the low levels of trust in science. However, even when information is easily accessible, people engage in biased and motivated reasoning: such as cognitive “glitches” that might be deeply rooted in our evolutionary past, stemming from adaptations for social persuasion and coalitional adaptations (refer to, e.g., Buss and von Hippel, 2018).

## Aim

Studies have identified a variety of correlates of conspiratorial thinking that could predict conspiratorial thinking. To further investigate the endorsement of COVID-19 conspiracy theories, we have proposed a model that includes various predictors of endorsement of COVID-19 conspiracy theories ranging from stable characteristics, such as demographics, through less stable general traits such as conservatism and overconfidence (Kruger and Dunning, 1999; Peterson et al., 2020), to relatively easily modifiable worldviews such as trust in science (Nadelson et al., 2014). We expected each of these variables to significantly contribute to the COVID-19 conspiracy’s endorsement. Specifically, we expected conservatism and overconfidence to be positive, and education, size of the place of residence, and trust in science and scientists to be negative predictors of beliefs in COVID-19 conspiracies. Furthermore, we were interested in the share of variance of believing in conspiracies that could be explained with each group of predictors above and beyond the preceding more stable characteristics in the model.

## Methods

### Participants and Procedure

Link to the online survey was distributed *via* mailing lists, social networks, and forums during the first 2 weeks of June 2020. This period was particularly convenient since the first wave had just ended, the number of daily new cases was very low, and the public was not convinced that the second wave of the pandemic was unavoidable. This context was fruitful in endorsing false beliefs about COVID-19, such as those claiming the disease is harmless or that it was engineered to make money from the vaccine.

On providing informed consent participants have proceeded to the instruments: (1) sociodemographic information, (2) syllogisms test and the estimation of one’s own performance, (3) trust in science and scientist inventory, (4) beliefs in COVID-19 conspiracy theory scale, and (5) conservatism scale.

A total of 859 participants clicked on the survey link, 837 agreed to participate, and 755 participants (413 female, 4 did not report gender) finished the survey. Their age ranged 16–69 (*M* = 27.86, *SD* = 11.73). Regarding education, 55.5% had elementary/high school grade, 21.7% BA degree, 17% MA/MS degree, and 5.8% PhD. While 58.3% lived in the cities, 41.7% lived in smaller towns or villages.

### Instruments

*Sociodemographic Information*. Participants reported gender, age, education level, and the population of their place of residence (8-point scale, ranging from below 2,000 to over 200,000 inhabitants).

*Conservatism*. A 12-item *Social and Economic Conservatism Scale* (SECS; Everett, 2013) measures conservatism, yet only the 7-item social conservatism subscale (abortion, army and national security, religion, traditional marriage, traditional values, family, and patriotism) was used for this study. Participants rated how they felt about each item on a scale of 0–100, with 0 = “very negative,” 100 = “very positive,” and 50 = “neither negative nor positive.” The subscale yielded good reliability (α = 0.85).

*Trust in Science and Scientist Inventory (Nadelson et al., 2014)*. A 21-item inventory was shortened to a 13-item form (Peterlin, 2019). Participants assessed their agreement with items, such as “*Scientific theories offer weak explanations*,” on a 5-point Likert scale (1 = extremely disagree; 5 = extremely agree). The reliability of the scale was satisfactory (α = 0.88).

*Syllogisms Test and Overconfidence in One’s Own Reasoning Abilities*. Participants were given 16 syllogisms to rate their validity, i.e., to respond to whether the conclusion is correct/logical, regardless of its factual correctness. The syllogisms were taken from the Markovits and Nantel (1989); 8 items or designed for this study (to ensure ecological validity, these syllogisms were pandemic-related; 8 items). A high correlation (0.79) among two groups of syllogisms has justified the use of a composite score. The overall Cronbach’s alpha was α = 0.86. Half of the syllogisms were logically valid. Syllogisms were balanced in a way that half of the valid conclusions were counterintuitive (logically, but not factually correct), while the other half were intuitive (logically and factually correct). Likewise, half of the incorrect conclusions were intuitive (logically and factually incorrect) and the other half were counterintuitive (factually correct, but logically incorrect). Their order was randomized. Finally, participants were asked about the number of syllogisms they believe to have solved correctly. The overconfidence was calculated as the difference between the estimated number of correct answers and the actual performance.

*Endorsement of COVID-19 Conspiracy Theories*. A 12-item scale was developed within this study to investigate the endorsement of various conspiracies regarding COVID-19. Participants indicated their agreement with the statements on a 5-point Likert scale (1 = extremely disagree; 5 = extremely agree). The scale yielded a high internal consistency (α = 0.90).

The full scale and descriptive statistics for *Endorsement of COVID-19 theories*, as well as the example in the syllogisms test, are available at the following link: https://osf.io/35zay/?view_only=b64c694b5cd94035a0cd61f244c0cac4.

## Results

Descriptive statistics and correlations are shown in Table 1. Scores on the syllogisms test covered the full range (min = 1, max = 16, *M* = 10.23, *SD* = 4.09). The correlation between the syllogisms test score and overconfidence score was significant (*r* = −0.57; *p* < 0.001), rendering the measure a good proxy of the Dunning-Kruger effect. For more details, refer to Appendix 1.

**TABLE 1:** Correlation matrix and descriptive statistics for all the variables in the model.

	*M*	*SD*	1	2	3	4	5	6	7
1 Endorsement of COVID-19 conspiracy theories	2.27	0.84	–						
2 Gender (1 = male; 2 = female)			0.12**	–					
3 Age	27.86	11.73	<0.01	-0.10*	–				
4 Education	2.71	0.97	-0.07	-0.11**	0.65**	–			
5 Residence size	6.40	2.28	-0.15**	-0.13**	0.10**	0.17**	–		
6 Conservatism	52.76	21.95	0.17**	0.07	-0.03	-0.12**	-0.10**	–	
7 Overconfidence	0.74	4.41	0.12**	-0.15**	0.12**	0.06	-0.09*	0.13**	–
8 Trust in science and scientists	3.78	0.71	-0.66**	-0.09*	<-0.01	0.04	0.16**	-0.16**	–0.09*

*p < 0.05; **p < 0.01.

The overall level of conspiracy endorsement was low. However, the total theoretical range was observed, and 17.4% of participants scored >3, indicating endorsement of at least some claims. Given their highly improbable nature (chips in vaccines, 5G spreading the virus, etc.) and the educational level of our sample, any level of endorsement would suggest susceptibility to conspiracies. The overall level of trust in science was rather high, which opens the possibility that a certain proportion of participants trusts science but lacks the tools to differentiate scientific from pseudoscientific claims.

Regarding our main hypothesis, we have predicted that sociodemographics, conservatism, overconfidence, and trust in science/scientists can significantly contribute to individual differences in the endorsement of COVID-19 conspiracies. Therefore, a three-step multiple regression analysis with belief in COVID-19 conspiracy theories as the criterion variable was conducted (Table 2).

**TABLE 2:** Summary of the three-step hierarchical regression analysis for variables predicting beliefs in COVID-19 conspiracy theories.

	Step 1			Step 2			Step 3		
Variable	*B*	*SE*	β	*B*	*SE*	β	*B*	*SE*	β
Gender (1 = male; 2 = female)	0.17	0.06	0.10**	0.19	0.06	0.12**	0.12	0.05	0.07*
Age	0.01	<0.01	0.09	<0.01	<0.01	0.06	<0.01	<0.01	0.03
Education	–0.07	0.04	–0.08	–0.05	0.04	–0.06	–0.03	0.03	–0.04
Residence size	–0.06	0.01	−0.16**	–0.05	0.01	−0.14**	–0.01	0.01	–0.04
Conservatism				0.01	<0.01	0.13**	<0.01	<0.01	0.05
Overconfidence				0.02	0.01	0.12**	0.01	0.01	0.06*
Trust in science and scientists							–0.75	0.03	−0.64**
*R* ^2^	0.05			0.08			0.46		
Δ*R*^2^	0.05**			0.03**			0.38**		

*p < 0.05; **p < 0.01.

Sociodemographics were entered in the first step and have accounted for 5% of the variation in the belief in COVID-19 conspiracies. These were more often endorsed by women and participants from smaller areas. Contrary to our predictions, education was not related to the endorsement of conspiracy theories. Conservatism and overconfidence in one’s reasoning (2nd step) have explained an additional 3% of the variance. This change in the explained variance was significant [*F*_(2, 675)_ = 12.5; *p* < 0.001]. More conservative and overconfident participants were also more prone to conspiracies. Finally, trust in science/scientists (3rd step) accounted for an additional 38% of the variance [*F*_(1, 674)_ = 481.5; *p* < 0.001], revealing that participants who trust science/scientists are less likely to endorse conspiracy theories. The final model accounted for 46% of the variance of believing in conspiracy theories with only gender, overconfidence, and trust in science being significant predictors.

Conservatism ceased to be a significant predictor in the third step, suggesting a potential mediating role of the trust in science/scientists in the relation between conservatism and conspiratorial thinking. To address this, we conducted the mediation analysis, which revealed that the relation between conservatism and conspiratorial beliefs is partially mediated by lower trust in science. The significance of the indirect effect was tested using 5,000 bootstrapped samples. The standardized indirect effect was significant (ab = 0.10: *p* < 0.01), as well as the standardized direct effect (*c*’ = 0.06: *p* < 0.05).

## Discussion

This study aimed to investigate the predictors of endorsement of the COVID-19 conspiracy theories grouped as (1) demographics (stable characteristics), (2) conservatism and overconfidence (less stable general traits), and (3) trust in science (easy-to-change worldview). The results of the HRA showed that women and participants from smaller areas were more likely to endorse COVID-19 conspiracy theories (5% of variance), more conservative and overconfident participants were more likely to endorse conspiracies (3% of variance), and participants who trust science are less likely to endorse COVID-19 conspiracies (38% of variance). Conservatism ceased to be a significant predictor in the third step of the analysis, suggesting a potential mediating role of the trust in science/scientists in the relationship between conservatism and conspiratorial thinking. The final model accounted for 46% of the variance of believing in COVID-19 conspiracy theories with gender, overconfidence in one’s own reasoning, and trust in science/scientists as significant predictors.

Our findings suggest that this widespread gullibility (Oliver and Wood, 2014b; Pew Research Center, 2020; an infodemic, as declared by the World Health Organization, 2020; Fuchs, 2021; refer to also European Commission, 2022) even among the formally educated population is partly driven by the overconfidence in one’s own reasoning. Similar to biased thinking, this self-deception in the form of *overestimating one’s own* abilities has an adaptive value: it protects one’s self-esteem, prevents the negative consequences of adverse events, protects mental health, and potentially helps in deceiving others (Trivers, 2000; von Hippel and Trivers, 2011). Nonetheless, the fact that overconfidence might have been evolutionarily selected as it offered fitness advantages does not imply that it is always beneficial. In fact, studies show that those with a higher risk of severe COVID-19 perceive a lower likelihood of infection and behave inconsistently with their elevated risk (Gassen et al., 2021).

The notion that overconfidence plays a significant role in the inability to differentiate between science and pseudoscience (the “I did my own research” dictum) tackles cognitive mechanisms, which might serve as a tool when planning interventions aimed at raising public understanding of complex phenomena such as the spread of a viral disease and mitigation methods. For example, in the context of the unintentional spreading of fake news on social media, it has been shown that nudging people to think about the accuracy of the shared content is a simple way to improve choices about what to share (Pennycook et al., 2020). An easy way for media platforms to implement these interventions would be providing quick feedback regarding the understanding of the read content, thus calibrating the reader’s perception of their own critical thinking skills before sharing the content alongside with own conclusions drawn from the shared content. Recent advances in psychological inoculation against fake news have shown that simple interventions such as warnings about fake news and pre-exposure to weakened doses of the techniques used in the production of fake news are effective in conferring psychological resistance against the endorsement of pseudoscientific narratives (e.g., Basol et al., 2020; Van Bavel et al., 2020; van der Linden et al., 2020). A gentle incentive to reevaluate one’s own beliefs and refine debunking skills even further might prove to be yet another “antigen” in psychological vaccines aimed at increasing the mind’s resistance to the viral fake news.

However, being overly optimistic about one’s knowledge and critical thinking skills is only a part of the equation. Our results suggest that this cognitive bias is often accompanied by a mistrust on health authorities and science in general, which explains the largest proportion of variance in the readiness to endorse pseudoscientific conspiratorial claims in our model. In fact, *conservatism*, which is often reported as an important predictor of conspiratorial thinking (van der Linden et al., 2020), was shown to lose its predictive power on the introduction of trust in science. The negative correlation we found between conservatism and trust in science hardly comes as a surprise considering the results of previous studies (e.g., Azevedo and Jost, 2021). For example, conservatives have been shown to score lower than liberals on the need for cognition, tolerance of ambiguity, cognitive reflection (Jost, 2017), actively open-minded thinking (Pennycook et al., 2020), and cognitive ability (Onraet et al., 2015). At the same time, they score higher on dogmatism, cognitive and perceptual rigidity, and personal need for order, structure, and closure (Jost, 2017) and have a more intuitive thinking style (Talhelm et al., 2015). All these abilities and traits are inherently related to the understanding of scientific processes and consequently in trusting science/scientists. Therefore, trust in science could have a mediating role in the relationship between conservatism and conspiratorial thinking, just as it does between authoritarianism and conspiratorial beliefs (Tonkoviæ et al., 2021). However, the mediation analysis showed that the relationship between conservatism and conspiratorial beliefs is only partially mediated by lower trust in science.

Worldwide attempts have been made to popularize science and explain complex phenomena to the general public and the possibility that such actions are a two-edged sword must be considered. Is it possible that the oversimplification of the rigorous process of drawing evidence-based conclusions has contributed to the banalization of scientific processes of formulating and testing hypotheses? Critical thinking is a valuable tool, but only when the thinker differentiates facts from fiction.

Since the beginning of the pandemic, some scientists have sent messages, adding fuel to the anti-science narrative. This is particularly unfortunate as once a conspiracy theory has taken its root, it is almost impossible to dislodge it, as the conspiracy turns those who argue against it into the “paid mercenaries of the system.” Thus, any attempts aimed at directly debunking such theories usually fail. A typical example is the Big Pharma conspiracy theory that flourished even before the COVID-19 pandemic, and what the research showed is that the fears over side effects and “unnatural” substances in vaccines are inextricably intertwined with the suspicion of the profit motive in healthcare (Paul et al., 2020). In the context of COVID-19, both conspiracy mentality and the anti-scientific sentiments are shown to negatively predict guideline adherence and vaccine intentions (Bertin et al., 2020; Marinthe et al., 2020; Hromatko et al., 2021; Santirocchi et al., 2022). Evidently, anti-scientific rhetoric bears serious consequences for both, individual health and health of the community.

This still leaves the question as to why we are currently facing such a rise in anti-scientific rhetoric. To trust in science, one must understand what the scientific process is. The educational system does not provide hands-on experience and a deeper understanding of what postulating and testing hypothesis entails. Pupils are presented with facts, only occasionally accompanied by a background story of a particular scientific discovery. Schools rarely provide a wider framework for understanding the scientific process itself: pupils are taught, on a theoretical level only, that the totality of human knowledge rises as a function of scientific progress, and that occasionally, a new discovery results in a paradigm shift. They are rarely sensitized to the fact that scientific endeavor is not a straightforward process, and that more often than not, a single study shall generate a small (if any) amount of new knowledge. Another point, which is also not systematically addressed, is the fact that the scientific consensus about any given matter is not instantly shattered when the findings of a single study are not in accordance with the findings of numerous others. For those members of the general public who are not more deeply acquainted with a certain area, conflicting results of scientific studies, being readily available, and disseminated *via* popular science outlets and social media may induce a feeling of mistrust in science/scientists. A possible solution would be the implementation of these basic tenets of the scientific process throughout all levels of education, not reserving them for graduate/expert programs only.

### Limitations of This Study

A key limitation inherent to online surveys is a non-representative sample, which might have been biased in terms of some relevant personality characteristics; participants higher on conscientiousness and agreeableness are more likely to participate in online studies. However, we have recruited a rather diverse sample in terms of age (age range of almost 50 years), a balanced proportion of men (44%) to women, and despite the disproportionately high number of educated participants, the overall education level ranged from elementary school only to Ph.D. Furthermore, 87.7% of participants answered all of the questions.

## Conclusion

Conspiratorial and biased thinking has been implicated in numerous fallacies, which are deeply rooted in the popular narrative. We have shown that the overestimation of one’s own reasoning, alongside the lack of trust in science, contributes to the endorsement of epistemically suspect beliefs regarding the pandemic. Such beliefs have the opportunity to incur damage on a large scale. Their direct debunking rarely yields success, so determining and addressing the precursors of such beliefs might prove to be more opportune. Given a large amount of variance in COVID-19-related conspiratorial thinking explained by the (mis)trust in science/scientists, it seems that restoring this trust is the most promising route for planning interventions. However, in the case of COVID-19, it might be too late for the implementation of such a large-scale top-down intervention. Another possible route involves a bottom-up mechanism and relies more on the individual psychological precursors, such as the self-deceptive nature of the assessments people make regarding their own abilities. Even though the share of variance of conspiratorial thinking explained by overconfidence is smaller than the one explained by mistrust in science, in the current situation when health officials are operating in the “damage-control” mode, such an intervention, alongside other psychological inoculation techniques, might offer an inexpensive and useful solution.

## Data Availability Statement

The raw data supporting the conclusions of this article will be made available by the authors, without undue reservation.

## Ethics Statement

The studies involving human participants were reviewed and approved by the Ethical Committee of the Department of Psychology, University of Zagreb. The patients/participants provided their written informed consent to participate in this study. Written informed consent was obtained from the individual(s) for the publication of any potentially identifiable images or data included in this article.

## Author Contributions

MT has analyzed the data. MT and IH have interpreted the data. AV has drafted the manuscript. All authors have designed the study and revised the final version.

## Conflict of Interest

The authors declare that the research was conducted in the absence of any commercial or financial relationships that could be construed as a potential conflict of interest.

## Publisher’s Note

All claims expressed in this article are solely those of the authors and do not necessarily represent those of their affiliated organizations, or those of the publisher, the editors and the reviewers. Any product that may be evaluated in this article, or claim that may be made by its manufacturer, is not guaranteed or endorsed by the publisher.

## Figures and Tables

**APPENDIX FIGURE 1:**
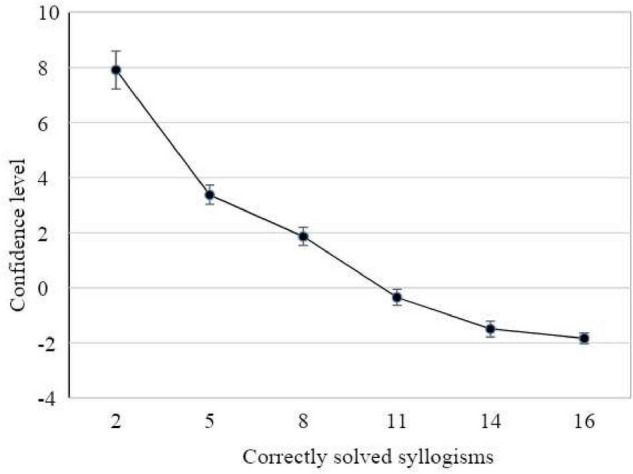
The relation of correctly solved syllogisms and the level of confidence (*N* = 755).

## Appendix

Appendix 1 | Description of the overconfidence measure. To explore whether the participants indeed expressed overconfidence, we conducted an ANOVA, with the number of correctly solved syllogisms as a source of variance, and the confidence level (the difference between the estimated number of correct responses and the actual number of correct responses) as the dependent variable. The results showed a significant effect [*F*_(5,749)_ = 75.78, *p* < 0.001] with *post-hoc* Scheffe’s tests indicating significant differences for all comparisons except between pairs of categories with the highest number of correctly solved syllogisms. As can be seen from Appendix Figure 1, the lower the scores on the syllogism test, the higher the (over)confidence. Participants with the average score (mean number of correctly solved syllogisms across participants was *M* = 10.2; *SD* = 4.09) made accurate estimates of their scores, while participants with above-average success tended to underestimate their scores.
